# Genotyping errors in a calibrated DNA register: implications for identification of individuals

**DOI:** 10.1186/1471-2156-12-36

**Published:** 2011-04-20

**Authors:** Øystein A Haaland, Kevin A Glover, Bjørghild B Seliussen, Hans J Skaug

**Affiliations:** 1 Department of Mathematics, University of Bergen, Johannes Brunsgate 12, 5008 Bergen, Norway; 2 Institute of Marine Research. P.O. Box 1870, Nordnes. N- 5817 Bergen, Norway

**Keywords:** Calibration, DNA register, genotyping error, microsatellite, minke whale, mixed logistic regression, wildlife

## Abstract

**Background:**

The use of DNA methods for the identification and management of natural resources is gaining importance. In the future, it is likely that DNA registers will play an increasing role in this development. Microsatellite markers have been the primary tool in ecological, medical and forensic genetics for the past two decades. However, these markers are characterized by genotyping errors, and display challenges with calibration between laboratories and genotyping platforms. The Norwegian minke whale DNA register (NMDR) contains individual genetic profiles at ten microsatellite loci for 6737 individuals captured in the period 1997-2008. These analyses have been conducted in four separate laboratories for nearly a decade, and offer a unique opportunity to examine genotyping errors and their consequences in an individual based DNA register. We re-genotyped 240 samples, and, for the first time, applied a mixed regression model to look at potentially confounding effects on genotyping errors.

**Results:**

The average genotyping error rate for the whole dataset was 0.013 per locus and 0.008 per allele. Errors were, however, not evenly distributed. A decreasing trend across time was apparent, along with a strong within-sample correlation, suggesting that error rates heavily depend on sample quality. In addition, some loci were more error prone than others. False allele size constituted 18 of 31 observed errors, and the remaining errors were ten false homozygotes (i.e., the *true *genotype was a heterozygote) and three false heterozygotes (i.e., the *true *genotype was a homozygote).

**Conclusions:**

To our knowledge, this study represents the first investigation of genotyping error rates in a wildlife DNA register, and the first application of mixed models to examine multiple effects of different factors influencing the genotyping quality. It was demonstrated that DNA registers accumulating data over time have the ability to maintain calibration and genotyping consistency, despite analyses being conducted on different genotyping platforms and in different laboratories. Although errors were detected, it is demonstrated that if the re-genotyping of individual samples is possible, these will have a minimal effect on the database's primary purpose, i.e., to perform individual identification.

## Background

Microsatellites, also known as short tandem repeats (STRs), are repeating sequences of DNA where the repeat motif includes 1-6 bases [1,2]. Variation in the number of repetitions within the sequence forms the basis of the alleles. Since their discovery in the 1980's, microsatellite DNA markers have been a prominent tool in ecological, medical and forensic genetics, among other things because of their high levels of variability, co-dominant inheritance, and abundance in most organisms [3-5].

Microsatellites are almost exclusively genotyped by amplification of the DNA sequence via the polymerase chain reaction, which is subsequently subject to electrophoresis and sized (i.e., length of repeat) in relation to known DNA fragments (i.e., the size standard). The relative migratory properties of the microsatellite fragment to the DNA size standard is influenced by a range of factors and is dependent on the conditions under which the electrophoresis is performed [6]. In part due to the way in which microsatellites are genotyped, this class of markers is prone to genotyping errors [7], which occur when the observed genotype does not correspond to the real genotype [8]. Genotyping errors in microsatellites cannot be avoided completely, and have a range of origins including scoring mistakes, contaminated multiplex assays, biochemical anomalies, and degenerated DNA samples [9]. Error rates in the range 0.005-0.01 per locus have frequently been reported in the literature [9]. Furthermore, error rates as low as 0.002 per locus are non-negligible, and may lead to false conclusions about, for example, confidence in assigned paternities [10].

The implementation of DNA based methods for the identification and management of wildlife resources represents a broad and rapidly growing field. In the future, it is likely that DNA-registers are going to become an increasingly important component of this development. For example, DNA-registers may contain information about animal pedigrees in living gene banks for conservation of endangered species, and monitor trade in wildlife [11,12]. DNA registers may be built upon a multitude of approaches and genetic markers, for example, relying upon allele frequencies for population identification [13], exact genotype profiles for individual identification [12], as well as sequence recognition for species identification in DNA barcoding [14]. Irrespective of primary purpose, a common feature of all DNA-registers is the fact that they accumulate data over time. This generates special challenges to data acquisition and quality, not least because developments in genotyping platforms and technology over time may cause calibration and continuity issues. Despite having similar genotyping equipment, different laboratories may still produce deviating allelic values for microsatellites on the same locus [6,15-17].

DNA-registers should be annotated with estimates of genotyping error rates from blinded experiments. For the purpose of matching profiles against a DNA-register, it is the across-profile error rate that is of importance, not the per-locus rates. If loci can be assumed independent, the former is given as(1)

when there are *L *loci, and *p*_*l *_is the error rate at locus *l*. However, when loci are positively correlated, i.e., the fact that an error occurs at one locus increases the error rate on other loci, the profile-wise error will exceed the value given by (1). We propose to account for this using a mixed regression model.

The Norwegian minke whale (*Balaenoptera acutorostrata*) DNA-register (NMDR) consists of individual DNA profiles from all whales captured legally (according to international law) by Norway since its establishment in 1997, and includes 6736 individuals up to the 2008 catch. The Norwegian whaling operations are controlled by the Norwegian Directorate of Fisheries, which also owns and operates the NMDR. In the period 1997-2008, four independent laboratories have had responsibility for the genotyping: Lab 1 from Canada (1997-2002), Lab 2 (2003-2005) and Lab 3 (2006), both from Iceland, and Lab 4 from Norway (2007-2008). All laboratories performed electrophoresis of the DNA fragments using Applied Biosystems genetic analyzers (Lab 1 = gel-based 377 machine, while laboratories 2-4 used capillary based machines). The initial analysis protocol was designed by the Norwegian forensic science institute using an ABI 377 genetic analyzer, and was based upon creating an allele size ladder for each of the markers. Each of the laboratories that have conducted genotyping for the register were first given sets of samples (up to 80 individuals) to calibrate their analyses against, and were also subject to and passed a blind calibration test with at least 20 known individuals prior to conducting analyses. Lab 1 started their analyses in the year 2000, thus, all 1668 individuals caught in 1999 or earlier were genotyped then. The NMDR was primarily established for forensic purposes, and low error rates have therefore been a high priority for the Norwegian government. The register includes >99.9% genotyping coverage for all markers in all individuals, which presents extra challenges when dealing with individual samples of low quality. Consequently, the NMDR provides a unique opportunity to check how the error rates in a high-quality DNA register has developed in an eight year period, and allows us to evaluate the precautions taken to deal with calibration issues between laboratories during a time of technological progress.

## Methods

### Genotyping and comparison to the NMDR

In order to check genotyping quality in the NMDR, 20 individual tissue samples from each year (1997-2008) were randomly selected for re-analysis. In total, 240 samples were subject to DNA isolation and amplification of 10 microsatellites that the register is based on: *GT509*, *GT310*, *GT211*, *GT575*, *GT023 *[18], *GATA098, GATA417, GATA028 *[19], *EV001PmG09074, EV037MnG09081 *[20]. Genotyping was conducted at the Institute of Marine Research in Bergen, Norway. This is the laboratory currently with responsibility for the register. In short, DNA was isolated from alcohol preserved muscle tissue in 96 well format using a commercial kit (Qiagen DNeasy). For Lab 1, Lab 2 and Lab 4 microsatellite DNA markers were amplified in three multiplex reactions, which was consistent with the original protocol. Lab 3 amplified fragments in two multiplexes for a single year class only. Full genotyping protocol is available [21].

After the first round of genotyping, individuals not providing full amplification of all markers were subject to PCR and electrophoresis again. Once samples were 100% genotyped, these data were compared to the genotypes stored in the DNA-register. A set of mismatch individuals (i.e., individuals where the genotype for at least one marker deviated between register and re-analysis here) was then subject to PCR and electrophoresis for a further 3-5 times in order to fully elucidate the genotype. All genotyping was manually inspected by two experienced researchers. After repeated analyses, genotypes still deviating from the DNA-register were considered as the *true *genotype, when, and only when, the re-run sample gave 100% consistent genotypes for the deviating marker in the 3-5 runs it was subject to in the present study. All genotyping errors detected within fulfilled this criterion, and no diffuse or inconsistent genotypes were accepted as the *true *genotype.

Six individual samples failed to amplify fragments for most of the markers, and were therefore excluded from the statistical analyses (two samples from 1997, one from 2008). The low and only partially amplified genotype peaks were indicative of poor DNA quality, probably caused by sub-optimal storage of those individual samples. While it is acknowledged that exclusion of these six samples may potentially decrease the overall estimate of genotyping errors if they were also of sub-optimal quality when originally genotyped, it was not possible to examine this here. Nevertheless, the primary purpose of this study, to test genotyping quality in a DNA register over time, through the application of a mixed regression model, was not compromised by exclusion of these six samples.

### Statistical methods

Logistic regression [22] was used to account for explanatory variables, such as locus effects, in the error rate estimation. It was not *a priori *assumed that errors occur independently, neither for the two alleles within a locus nor across loci. Errors are said to be independently distributed within a locus of an individual, if the event that the first allele (gene copy) is erroneously recorded does not affect the probability of an error occurring at the second allele of that same locus. Such independence yields the relationship(2)

where *p*_*l *_is the per-locus error rate, and *p*_*a *_is the per-allele (gene copy) error rate. The per-locus error rate is the probability that at least one error occurs at a locus of an individual. The data was analyzed both with respect to *p*_*l *_and *p*_*a*_, but no differences of consequence were detected. We shall therefore focus on *p*_*l*_, as it is the most commonly used metric among the two [23,24].

Mixed (regression) models are commonly used to relate correlated observations to explanatory variables [22]. We distinguished between fixed effects (LAB, LOCUS and YEAR), which affected all individuals in the population equally, and random effects which were individual specific effects. The fixed effects LAB and LOCUS were taken to be factors (ANOVA type) representing systematic differences between laboratories and loci caused by, e.g., diverging protocols and differing ranges of allele sizes. One parameter was estimated per level of the factors. The fixed effect YEAR was a continuous covariate (regression type), expressing the effect of a steady advance in technology. Two random effects, IND (individual sample) and MP:IND (intersection between individual and multiplex assay), were considered. IND affected the error rate at all loci equally, while MP:IND affected only those loci (within a particular individual) ran together on a multiplex. They expressed discrepancies due to varying sample quality and mishandling of equipment, respectively, and were taken to be normally distributed with mean zero and standard deviations *σ*_*IND *_and *σ*_*MP:IND*_, respectively. Low values of *σ*_*IND *_and *σ*_*MP:IND *_means little difference between individuals, and hence little impact on the error rates, because the random effects are centered at zero. Due to the facts that no laboratory has been responsible for the analyses more than three years (Table 1), and that not all labs were assessed each year in the study (Table 1), YEAR and LAB are confounded covariates, and consequently never appear in the same model (Table 2).

**Table 1:** Empirical estimates of error rates by laboratory.

			LOCUS		ALLELE		PRED
			
Lab (country)	Period	Sample size	***p***_***l***_	**SD(*p***_***l***_**)**	***p***_***a***_	**SD(*p***_***a***_)	
Lab 1 (Canada)	97-02	116 (120)	0.02373	0.00443	0.01422	0.00246	0.02825
Lab 2 (Iceland)	03-05	60	0.00500	0.00288	0.00333	0.00166	0.00666
Lab 3 (Iceland)	06	19 (20)	0	-	0	-	0
Lab 4 (Norway)	07-08	39 (40)	0.00256	0.00256	0.00128	0.00128	0.00256
Total	97-08	234 (240)	0.01325	0.00236	0.00812	0.00131	0.01617

The column "Period" shows the catch period for which each laboratory had responsibility for the NMDR. Except for the catches from 1997-98, which were genotyped in the year 2000, all individuals were genotyped the year following capture. The column "Sample size" contains the number of successfully regenotyped individuals from the laboratories, with the total number of regenotyping attempts in parenthesis. Initially 20 samples were selected from each year, but 6 samples had to be thrown out because of amplification failure. Locus *EV001 *was included for all probabilities of error in this table. In columns labeled LOCUS and ALLELE, *p*_*l *_and *p*_*a *_are the mean error rates (number of errors detected divided by number of loci/alleles), and SD(p) the corresponding empirical standard deviations. The column labeled PRED contains the predicted values, , of *p*_*l*_, calculated from *p*_*a *_using (2).

**Table 2:** Candidate model set for NMDR study.

Model	Fixed	Rand	*σ*	AIC	p	SD(p)	CV(p)	W
M1	LOCUS + YEAR	IND	1.35	295.7	0.0774	0.0214	0.28	5.76e-1

M2	YEAR	IND	1.24	297.5	0.0243	0.0045	0.19	2.34e-1

M3	LAB + LOCUS	IND	1.43	299.3	0.0514			9.52e-2
	Lab1				0.0823	0.0242	0.29	
	Lab2				0.0185	0.0088	0.48	
	Lab4				0.0102	0.0089	0.87	

M4	LAB	IND	1.32	301.0	0.0162			4.07e-2
	Lab1				0.0261	0.0045	0.17	
	Lab2				0.0054	0.0024	0.44	
	Lab4				0.0029	0.0025	0.86	

M5	LOCUS + YEAR	-	-	303.1	0.0768	0.0239	0.29	1.42e-2

M6	LOCUS + YEAR	MP:IND	1.22	303.5	0.0771	0.0237	0.33	1.17e-2

M7	YEAR	-	-	303.6	0.024	0.0046	0.2	1.11e-2

M8	YEAR	MP:IND	1.13	304.0	0.0242	0.0047	0.2	9.08e-3

M9	LAB + LOCUS	-	-	307.1	0.0511			1.92e-3
	Lab1				0.0822	0.0263	0.32	
	Lab2				0.0182	0.0118	0.65	
	Lab4				0.0094	0.0097	1.04	

M10	LAB + LOCUS	MP:IND	1.31	307.2	0.0516			1.83e-3
	Lab1				0.0828	0.0405	0.49	
	Lab2				0.0193	0.0172	0.89	
	Lab4				0.0102	0.0131	1.28	

M11	LAB	-	-	307.5	0.016			1.58e-3
	Lab1				0.0259	0.0048	0.19	
	Lab2				0.0056	0.0032	0.58	
	Lab4				0.0028	0.0031	1.08	

M12	LAB	MP:IND	1.20	307.9	0.0162			1.29e-3
	Lab1				0.0261	0.038	0.15	
	Lab2				0.0056	0.081	1.44	
	Lab4				0.0028	0.062	2.15	

M13	LOCUS	IND	1.52	308.3	0.0513	0.0129	0.25	1.06e-3

M14	-	IND	1.41	309.9	0.016	0.0026	0.17	4.75e-4

M15	LOCUS	MP:IND	1.50	318.3	0.0523	0.0057	0.11	7.12e-6

M16	-	MP:IND	1.36	319.1	0.0162	0.0055	0.36	4.78e-6

M17	LOCUS	-	-	319.5	0.0512	0.0156	0.3	3.91e-6

M18	-	-	-	319.8	0.016	0.0030	0.2	3.37e-6

Model fit and estimated error rates for different covariate models sorted according to the AIC score (column five). "*σ*" contains the standard deviations of the random effects. The values in "p" are the probabilities that an error occurs at locus *GATA417 *in year 2001. Whenever LAB is included in the model, weighted means and laboratory specific error rates are given (Lab 1, Lab 2, Lab 4). "SD(p)" and "CV(p)" are the bootstrap based standard deviations and coefficients of variance of p. The column "W" contains the Akaike weights of the models.

Because the response variable was dichotomous (i.e., error vs. not error), a logistic regression model was used ([22], p 274). Denote by *p*_*il *_the probability that an error occurs at locus *l *of individual *i*. A logistic map was used to model the effect of the explanatory variables on *p*_*il*_,(3)

where *η*_*il *_is a linear predictor, expressed in Witkinson-Rogers notation as(4)

This is referred to as the full model. The notation in (4) is standard in, e.g., R, and is a simplification of , where the *β*'s are regression parameters, the *b*'s are normally distributed random variables and *lab*_*i *_denotes the laboratory of the *i*'th individual. There were in total 215 random *b*^*IND *^parameters, and 645 *b*^*MP:IND *^parameters. Submodels, with individual terms in (4) removed, were also fitted to the data. For models without "LOCUS" the error rate does not depend on the index "*l*", and we hence write *p*_*i*_. In the absence of both IND and MP:IND, the model reduced to an ordinary logistic regression with no individual specific effects. The AIC criterion [25] was used to balance goodness of fit and parsimony, and Akaike weights ([26], p 75) were calculated. The Akaike weight for the *k*'th model is

where Δ_*k *_= *AIC*_*k *_- *AIC*_*min*_, and *AIC*_*min *_is the smallest AIC-value. For models where individual specific effects (IND or MP:IND) were included, the probability (3) was averaged over individual effects using Monte Carlo simulations (Appendix A).

All models were fit using the R package "glmmADMB" which builds on the software package AD Model Builder (AD Model Builder project, 2009). Because glmmADMB could not fit models with more than one random effect present simultaneously, we relied on SAS PROC GLIMMIX in such situations. A bootstrap approach was used to obtain the standard deviations of the error rate, *p*, given by (3). Individuals were randomly sampled from the data with replacement to create 500 bootstrap datasets. For each of these the model was refit, and finally, standard deviations were calculated empirically across the 500 bootstrap replica. Due to the use of sampling with replacement, the number of genotyping errors differed across the replica, and also from the original data set. When fitting a model using bootstrap replica which happened to have few genotyping errors, the software sometimes produced warnings indicating non-convergence of the numerical method. However, such warnings were rare (1.6% for M1, 0.6% for M10, 3.6% for M15 and zero for the others), and only modestly affected the estimated standard deviations. To obtain an upper bound for the effect of the non-convergence, we counted all error estimates from bootstrap replica producing warnings as zero. Results for M1 are showed in Table 3.

**Table 3:** Estimates of (per locus) error rates for best fitting model (M1).

Locus	p	SD(p)	CV(p)
*GATA417*	0.0774	0.0214 (0.0241)	0.28
*EV037*	0.0283	0.0112 (0.0122)	0.41
*GATA028*	0.0283	0.0130 (0.0134)	0.46
*GT575*	0.0213	0.0110 (0.0112)	0.52
*GT509*	0.0141	0.0093 (0.0095)	0.66
*GT310*	0.0141	0.0101 (0.0102)	0.71
*GT211*	0.0141	0.0083 (0.0085)	0.59
*GT023*	0.0141	0.0091 (0.0092)	0.64
*GATA098*	0.0071	0.0084 (0.0084)	1.19
*EV001*	0	0	-

The values in "p" are the probabilities that an error occurs at a particular locus in year 2001. "SD(p)" and "CV(p)" are the bootstrap based standard deviations and coefficients of variance of p. In parenthesis are the "upper limit" standard deviations (i.e., we included bootstrap replica which produced warning messages as having zero errors).

## Results

### Descriptive statistics

The mean genotyping error rate varied both between laboratories and from year to year (Table 1). For the analyses of the catches from 2007-8, conducted by Lab 4, the mean error rate was approximately a ninth of what was found for the oldest analyses, conducted by Lab 1. Lab 3 and locus *EV001 *had error rates of zero, and were therefore left out of the subsequent statistical analyses, as these data did not provide information about other parameters than those associated with the respective factor levels (Lab 3 and locus *EV001*). Using the *p*_*a *_estimated from the data, (2) predicted the corresponding value of *p*_*l *_to three decimal points for Lab 4 (Table 1). On the other hand, for Lab 2 and Lab 1 the predicted values of *p*_*l *_(still using the estimated *p*_*a *_and (2)) were off by 33% and 19%, respectively (Table 1), indicating the presence of individual or multiplex assay effects, which is consistent with [8]. False allele size constituted 18 of the 31 errors (Table 4). A third of those were incorrectly called at both gene copies, again pointing in the direction of the presence of individual sample or multiplex effects. The remaining errors were ten false homozygotes (i.e., the *true *genpotype was a heterozygote) and three false heterozygotes (i.e., the *true *genotype was a homozygote).

**Table 4:** Summary of genotyping errors observed in the NMDR according to laboratory.

	**Lab 1**			**Lab 2**			**Lab 4**		
**Type of error**	**Total**	**= 1 bp**	**>1 bp**	**Total**	**= 1 bp**	**>1 bp**	**Total**	**= 1 bp**	**>1 bp**
			
Single false allele size	**10**	7	3	**2**	0	2	**0**	0	0
Double false allele size	**6**	1	5	**0**	0	0	**0**	0	0
False homozygote	**9**	5	4	**1**	1	0	**0**	0	0
False heterozygote	**2**	0	2	**0**	0	0	**1**	0	1

Columns labeled "Total" contain the total number of errors of a specific kind for each laboratory. False allele size is when an allele was erroneously sized compared to the *true *size for single and double alleles. " = 1 bp" denotes that the erroneously called allele is a distance one from the *true *allele, whereas ">1 bp" means that the distance was greater than one. False homozygote is where the *true *genotype was a heterozygote, and false heterozygote is where the *true *genotype was a homozygote.

### Model selection

Models were arranged according to their AIC value, which balances goodness of fit against model complexity (Table 2). The covariate YEAR is confounded with LAB, but had a better fit than LAB according to the AIC criterion. Models including YEAR had a combined Akaike weight of 0.86, while the Akaike weights of models containing LAB only summed to 0.14 (Table 2). Part of the reason is that YEAR gave a more parsimonious representation (1 extra parameter) of the fact that technology has improved over time than did LAB (2 extra parameters). The addition of LOCUS to a model always improved the fit (e.g., M1 vs. M2, Table 2). The combined Akaike weight of models including IND was 0.95, whereas models featuring MP:IND had Akaike weights summing to 0.024 (Table 2). In all cases where IND and MP:IND were present in the same model, *σ*_*MP:IND *_was estimated to be equal to zero, reducing the model to one including only IND. Therefore, models including both random effects were omitted from further consideration.

### Error rates

The non-model based mean error rate across all labs and years, including locus *EV001 *and Lab 3, was 0.013 per locus and 0.008 per allele (Table 1). Excluding *EV001 *and Lab 3, the mean error rate was 0.016 per locus. Under the best fitting model (M1), the across-locus error rate was 0.022 in the middle of the period (year 2001). The locus with the highest error rate was *GATA417*, at which the rate (0.077) was almost three times as high as at any other locus (Table 3). In 2004 and 2008 the error rates for *GATA417 *had dropped to 0.032 and 0.008, still according to M1.

Models containing the same fixed effects yielded similar error rate estimates, regardless of the choice of random effects (e.g., M1, M5 and M6, Table 2), so to compare laboratories, we discuss models including LAB and LOCUS apart from models featuring only LAB. In both scenarios the error rate at Lab 1 was several times higher than at the other laboratories, and Lab 4 always had lower error rates than Lab 2 (e.g., M3, Table 2). This is consistent with the estimated regression coefficient for YEAR translating to a decreasing annual trend. Note that, in Table 2, *p *was calculated for *GATA417*, where errors were abundant, and in the year 2001, which is why *p *rose when LOCUS or YEAR was added to a model (e.g., M14 vs. M2, and M2 vs. M1, Table 2).

Our suspicion that errors tend to accumulate within individuals was strengthened by *σ *being greater than zero in all models featuring random effects (Table 2). The importance of this fact becomes obvious when considering how it affects the probability of more errors occurring at individuals with at least one error. Due to the dependence between loci, we expect more than one in five such individuals to have at least two errors, whereas independence would yield less than one in ten (Table 5).

**Table 5:** Multilocus error rates.

	*P(E > 0)*	*P(E = 1)*	*P(E > 1)*	*P(E > 1|E > 0)*
Dep.	17.0	13.4	3.60	21.2

Ind.	20.1	18.3	1.77	8.84

Probability of having *E *errors in a 10 loci profile for model M1. The top row (Dep.) assumes dependence between the loci within an individual, and the bottom row (Ind.) assumes independence. *P(E > 1|E > 0) *is the conditional probability of having two or more errors given that an error occurs.

**Figure 1:**
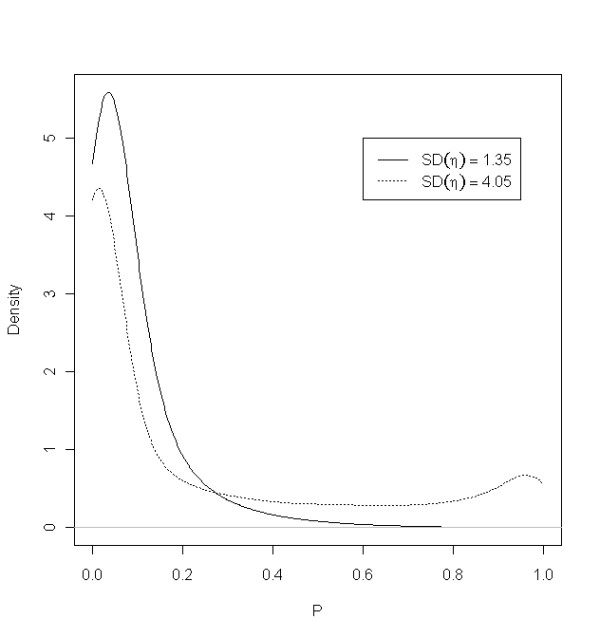
**Probability densities for error rates, *p*, for model M1 (*SD *(*η*) = 1.35) and a model with a three times larger standard deviation (*SD *(*η*) = 4.05)**.

In the mixed model framework, the per locus error probability varies among individuals according to a distribution (Figure 1). For the estimated value of *σ *this distribution was unimodal, but skewed to the right. Because the random effect was shared among loci, individuals in the right hand tail of the distribution were error prone at all loci. In an artificial experiment, where *σ *was increased by a factor of three, the population distribution became bimodal, with the two groups of individuals being 1) those with almost zero error rate 2) those with *p*_*il *_> 0.50 (Figure 1).

Table 6 shows the mean allele lengths for each marker, and the corresponding coefficients for model M1 (Table 2), sorted according to mean error rate. *GATA417 *was by far the most error prone locus. At the most ancient laboratory, Lab 1, there were nine occurrences of the *true *genotype, 221, being erroneously recorded as 220 (data not shown) for *GATA417*. Both 220 and 221 were present on the allelic ladder. Apart from those, *GATA417 *had two errors.

**Table 6:** Error rates and allele lengths.

Parameter	Coef	SD(coef)	***A***_***max***_		
*GATA417*	1.59	0.38	252	223	0.0512
*EV037*	0.40	0.52	211	203	0.0186
*GATA028*	0.40	0.52	223	202	0.0186
*GT575*	0.09	0.58	166	155	0.0140
*GT509*	-0.35	0.68	217	203	0.0093
*GT310*	-0.35	0.68	125	117	0.0093
*GT211*	-0.35	0.68	116	106	0.0093
*GT023*	-0.35	0.68	115	103	0.0093
*GATA098*	-1.08	0.93	107	93	0.0047
*EV001*	-	-	175	153	0
YEAR	-0.36	0.11	-	-	-
Intercept	-4.74	0.45	-	-	-
VAR(IND)	1.81	0.94	-	-	-

The second column is the values of the coefficients in M1 (Table 2), and the third is their standard deviations. *A*_*max *_is the length of the largest allele, and  is calculated using the population allele frequencies.  is the mean error rate at the locus. *EV001 *was not in the analyses, and has therefore no coefficient in M1. Its correct value would have been -∞.

## Discussion

To our knowledge, this study represents the first investigation of genotyping error rates in a wildlife DNA register, and the first application of mixed models to examine multiple effects of different factors influencing the genotyping quality, such as time, microsatellite marker and sample quality.

A major challenge with microsatellite data sets is sharing data between laboratories, and comparing data from different analytical platforms. Despite the importance of these challenges, systematic shifts of allelic scores have, with few exceptions, not been thoroughly examined (e.g., [6,15,16]). Furthermore, to our knowledge no studies have investigated how calibration between laboratories over time influences the ability to produce calibrated data. Although nine *true *values of 221 were recorded as 220 at *GATA417 *and Lab 1, we did not detect any systematic shift of allelic values for any of the markers implemented in the NMDR. This was despite the fact that the analyses were conducted in four separate laboratories in three countries, and over a period almost stretching a decade. We conclude that even though the genotyping for the NMDR has been conducted by several laboratories, and during a period in which genotyping platforms have displayed significant technological changes from gel to capillary based electrophoresis instruments, systematic genotyping errors due to allele size calibration were not present. This demonstrates the importance of calibrating genotyping scoring between laboratories, in addition to conducting blind proficiency tests prior to new laboratories overtaking an existing DNA register, as was performed for the NMDR.

Assuming genotyping errors disperse identically and independently (or almost independently) across the markers within an individual can be convenient when dealing with calculations regarding the genotyping error rate. Among studies utilizing simulations, it is therefore a common simplification [10,24,27,28]. However, such an assumption is often not realistic [7,27,29]. This is well illustrated by a study of genotyping errors in 510 loci [8]. In that study, ten errors were detected, and all occurred in the same individual. Within-individual dependencies like this can easily be modeled by increasing the standard deviation of the random effects MP:IND and IND in the mixed models (4).

Because all individual whales caught by Norway are required to be genotyped at all markers in the NMDR, samples of questionable quality cannot be disregarded or left as missing data, as is possible in many studies [8,30]. Despite this fact, the overall error rate of 0.013 per locus in the NMDR is in concordance with the published literature on microsatellites from tissue samples [7-9,31]. Still, the inclusion of bad samples in the analysis is an error source beyond the control of any laboratory, and contributes to the standard deviation of IND, *σ*_*IND*_, representing the effect of variability in sample quality between individuals, being greater than zero in all models featuring IND (Table 2).

We initially investigated an interaction effect between individual and multiplex (MP:IND), but it turned out to be superfluous if a model also contained an individual random effect (IND). Further, the inclusion of MP:IND alone in a model not containing IND did not affect the model fit much (Table 2). This leads to the conclusion that sample quality was dominating over the mishandling of multiplex assays as a source of errors, but the same conclusion may not apply in other studies.

Among the fixed effects, YEAR was the most important. Because LAB and YEAR were confounded, we were unable to assess their individual impacts on the genotyping error rate. However, models including YEAR had the best AIC scores (Table 2). This can partly be explained by LAB being a less parsimonious representation of technological and procedural advances than YEAR, and partly by models including YEAR being closer to the data than models including LAB (Table 2). Examining the difference between technological and procedural advances, we conclude that the considerable impact of sample quality (IND) relative to that of the mishandling of multiplex assays (MP:IND) implies that the importance of YEAR is mostly due to progress regarding apparatus. Although the complete eradication of genotyping errors seems unlikely, we have documented a positive development taking place over the last decade in the NMDR.

In addition to the time aspect, a large variation between the genotyping error rate on different markers was detected (Table 6). Such variation was expected [29], and was the reason for the positive impact of LOCUS on the model fit. It is also known that larger alleles may be more prone to genotyping errors than shorter ones [32-34], which is consistent with the trend seen here (Table 6). The relationship between allelic size and error rate is not deterministic however, as illustrated by locus *EV001 *harboring zero errors (Table 6).

Initially the R function "lmer" was used to fit the mixed models. However, due to convergence problems on the bootstrap datasets, we switched to the R package "glmmADMB" which turned out to be more numerically robust, but had the limitation that only a single random effect can be included at a time. This is the reason why models including both the factors IND and MP:IND were run in SAS.

Since the beginning of the millennium, the number of peer reviewed articles mentioning genotyping errors has drastically increased [9]. It has been discussed how to best obtain estimates for genotyping error rates [23,31,35], reduce the number of errors [7,8] and statistically handle the uncertainty necessarily accompanying errors [10,27]. We have focused on how to accurately model the genotyping error rate. This is important both in order to understand the underlying mechanisms concerning errors, and to be able to use data for purposes of statistical inference.

The presence of genotyping errors weakens the ability to accurately match individual samples to a DNA register. On average there is a 17% chance of a mismatch between a true multilocus genotype and the corresponding genotype in the NMDR for data accumulated over a decade (Table 5). Tissue samples from all individuals are stored, so it is possible to analyze them again, and thereby correct prospective errors. This is handy, e.g., in a juridical setting [12], where a high confidence in the validity of the genotypes is imperative in order to take legal action, and only few samples are involved. The use of genetic tagging to obtain abundance estimates [36-39] and to monitor populations [19,40-42] is widespread. For such applications, reanalyzing all close mismatches (sample matching at all but a few markers) may not be feasible due to financial or other reasons. If a rule is applied to allow for close mismatches to count as a recapture, the within-individual dependence structure described by *σ *is of great importance. Assuming independence (*σ *= 0) when it is not the case, the probability of more errors occurring at individuals harboring at least one error will potentially be grossly underestimated (Table 5). Genotyping errors may also strongly influence the outcome of parentage analysis [10,43]. As with individual identification, one can compensate by assigning parentage even if a candidate parent-offspring pair does not have at least one allele in common at all markers [44,45]. A somewhat related matter is the degree of relatedness represented by LOD score [46,47] used in, e.g., [48-50] to do inference about population structure and size from identified close kin. In both cases it matters whether few individuals contain many genotyping errors or the errors are more evenly spread.

## Conclusions

Microsatellite DNA markers with associated genotyping quality issues can be challenging to handle, especially in the context of a DNA register that requires accurate data over many years. Nevertheless, results of the present study have demonstrated that accurate, calibrated and reproducible genotypic data are possible to achieve despite conducting analyses over a number of years and a number of analytical platforms. In addition, the mixed models approach implemented in the present study has provided further clarification of how genotyping errors occur. Once an error is present at one marker of an individual sample, it is more likely that other markers of that same individual have also been erroneously recorded. This has important consequences when making inference on individual matches, parental assignment, or degree of relatedness.

## Authors' contributions

All authors have read and approved the final manuscript, contributed to the design of the study, and contributed to interpretation of data. BBS and KAG conducted genetic analyses while ØAH and HJS conducted statistical modeling of error rates. ØAH coordinated writing of the manuscript to which all authors contributed.

## Appendix A

In this appendix our first goal is to explain how the individual specific error probability (3) is averaged over the individual effects. Letting *p*_*l *_= *P *(Error at locus 1), we get(5)

where *f*_*l*_(η) is the Gaussian probability distribution function at locus *l *with mean *μ*_*l *_*= LAB + LOCUS*_*l *_*+ YEAR*, and variance . Drawing *N *different *η*_*il *_(*i *= 1,...,*N*) from *f*_*l *_(η), (5) may be approximated by the Monte Carlo estimate(6)

A second goal of this appendix is to derive Table 5. Begin by letting *E *be the number of errors at an individual across all loci. For an individual *i*(7)

and,(8)

where *p*_*il *_is as in (6). Assuming that errors are independently distributed across the loci of that same individual, we may simply substitute *p*_*il *_in (7) and (8) with *p*_*l *_from (6), to get the population means

as already seen in (1), and

Without the independence assumption, the calculations require the simultaneous consideration of all nine loci for each of the *N *simulated individuals. Hence, Monte Carlo estimation gives

with *P*_*i *_(E > 0) given by (7), and

with *P*_*i *_(E = 1) given by (8). In both the independence- and dependence scenario

and

## References

[B1] Choudhary M Strassmann JE Solis CR Queller DC Microsatellite Variation in a Social Insect Biochemical Genetics 1993 31 1-2 87 96 10.1007/BF02399822 8471026

[B2] Litt M Luty JA A Hypervariable Microsatellite Revealed by Invitro Amplification of a Dinucleotide Repeat within the Cardiac-Muscle Actin Gene American Journal of Human Genetics 1989 44 3 397 401 2563634 PMC1715430

[B3] Jarne P Lagoda PJL Microsatellites, from molecules to populations and back Trends in Ecology & Evolution 1996 11 10 424 429 10.1016/0169-5347(96)10049-5 21237902

[B4] Luikart G England PR Statistical analysis of microsatellite DNA data Trends in Ecology & Evolution 1999 14 7 253 256 10.1016/S0169-5347(99)01632-8 10370259

[B5] Queller DC Strassmann JE Hughes CR Microsatellites and Kinship Trends in Ecology & Evolution 1993 8 8 285 & 10.1016/0169-5347(93)90256-O 21236170

[B6] Pasqualotto AC Denning DW Anderson MJ A cautionary tale: Lack of consistency in allele sizes between two laboratories for a published multilocus microsatellite typing system Journal of Clinical Microbiology 2007 45 2 522 528 10.1128/JCM.02136-06 17166958 PMC1829014

[B7] Hoffman JI Amos W Microsatellite genotyping errors: detection approaches, common sources and consequences for paternal exclusion Molecular Ecology 2005 14 2 599 612 15660949 10.1111/j.1365-294X.2004.02419.x

[B8] Bonin A Bellemain E Bronken Eidesen P Pompanon F Brochman C Taberlet P How to track and assess genotyping errors in population genetics studies Molecular Ecology 2004 13 11 3261 3273 10.1111/j.1365-294X.2004.02346.x 15487987

[B9] Pompanon F Bonin A Bellemain E Taberlet P Genotyping errors: Causes, consequences and solutions Nature Reviews Genetics 2005 6 11 847 859 10.1038/nrg1707 16304600

[B10] Marshall TC Marshall TC Slate J Kruuk LEB Pemberton JM Statistical confidence for likelihood-based paternity inference in natural populations Molecular Ecology 1998 7 5 639 655 10.1046/j.1365-294x.1998.00374.x 9633105

[B11] Baker CS Steel D Choi Y Lee H Kim KS Choi SK Ma Y-U Hambleton C Psihoyos L Brownell RL Funahashi N Genetic evidence of illegal trade in protected whales links Japan with the US and South Korea Biology letters 2010 6 5 647 650 10.1098/rsbl.2010.0239 20392716 PMC2936163

[B12] Palsboll PJ Berube M Skaug HJ Raymakers C DNA registers of legally obtained wildlife and derived products as means to identify illegal takes Conservation Biology 2006 20 4 1284 1293 10.1111/j.1523-1739.2006.00429.x 16922244

[B13] Withler RE Candy JR Beacham TD Miller KM Forensic DNA analysis of Pacific salmonid samples for species and stock identification Environmental Biology of Fishes 2004 69 1-4 275 285

[B14] Hebert PDN Cywinska A Ball SL deWaard JR Biological identifications through DNA barcodes Proceedings of the Royal Society of London Series B-Biological Sciences 2003 270 1512 313 321 10.1098/rspb.2002.2218 PMC1691236 12614582

[B15] Baric S Monschein S Hofer M Grill D Dalla Via J Comparability of genotyping data obtained by different procedures an inter-laboratory survey Journal of Horticultural Science & Biotechnology 2008 83 2 183 190

[B16] de Valk HA Meis JFGM Bretagne S Costa J-M Lasker BA Balajee SA Pasqualotto AC Anderson MJ Alcazar-Fuoli L Klaassen CHW Interlaboratory reproducibility of a microsatellite-based typing assay for Aspergillus fumigatus through the use of allelic ladders: proof of concept Clin Microbial Infect 2009 15 180 187 10.1111/j.1469-0691.2008.02656.x 19154486

[B17] Ellis JS Gilbey J Armstrong A Balstad T Cauwelier E Cherbonnel C Consuegra S Coughlan J Cross TF Crozier W Dillane E Ensing D Garcia de Leaniz C Garcia-Vazquez E Griffiths AM Hindar K Hjorleifsdottir S Knox D Machado-Schiaffino G McGinnity P Meldrup D Nielsen EE Olafsson K Primmer CR Prodohl P Stradmeyer L Vaha JP Verspoor E Wennevik V Stevens JR Microsatellite standardization and evaluation of genotyping error in a large multi-partner research programme for conservation of Atlantic salmon (Salmo salar L.) Genetica 2011 139 3 353 67 10.1007/s10709-011-9554-4 21279823 PMC3059809

[B18] Berube M Jørgensen H McEwing R Palsbøll PJ Polymorphic di-nucleotide microsatellite loci isolated from the humpback whale, Megaptera novaeangliae Molecular Ecology 2000 9 12 2181 3 10.1046/j.1365-294X.2000.105315.x 11123643

[B19] Palsboll PJ Allen J Berube M Clapham PJ Feddersen TP Hammond PS Hudson RS Jørgensen H Katona S Larsen AH Larsen F Lien J Sears R Smith T Sponer R Stevick P Øien N Genetic tagging of humpback whales Nature 1997 388 6644 767 769 10.1038/42005 9285587

[B20] Valsecchi E Amos W Microsatellite markers for the study of cetacean populations Molecular Ecology 1996 5 1 151 156 10.1111/j.1365-294X.1996.tb00301.x 9147690

[B21] Glover KA Kanda N Haug T Pastene LA Øien N Goto M Seliussen BB Skaug HJ Migration of Antarctic Minke Whales to the Arctic PLos ONE 2010 5 12 e15197 10.1371/journal.pone.0015197 21203557 PMC3008685

[B22] Pinheiro JC Bates DM Chambers J, Eddy W, Härdle W, Sheater S, Tierney L Mixed-Effects Models in S and S-plus Statistics and Computing 2000 Springer

[B23] Broquet T Petit E Quantifying genotyping errors in noninvasive population genetics Molecular Ecology 2004 13 11 3601 3608 10.1111/j.1365-294X.2004.02352.x 15488016

[B24] Johnson PCD Haydon DT Maximum-likelihood estimation of allelic dropout and false allele error rates from Microsatellite genotypes in the absence of reference data Genetics 2007 175 2 827 842 10.1534/genetics.106.064618 17179070 PMC1800638

[B25] Akaike H New Look at Statistical-Model Identification Ieee Transactions on Automatic Control 1974 Ac19 6 716 723

[B26] Burnham KP Anderson DR Model selection and multi-model inference: A practical information-theoretic approach 2002 2 New York, NY: Springer-Verlag 488

[B27] Paetkau D An empirical exploration of data quality in DNA-based population inventories Molecular Ecology 2003 12 6 1375 1387 10.1046/j.1365-294X.2003.01820.x 12755868

[B28] Zhang HM Stern H Assessment of ancestry probabilities in the presence of genotyping errors Theoretical and Applied Genetics 2006 112 3 472 482 10.1007/s00122-005-0148-3 16307226

[B29] Mitchell AA Cutler DJ Chakravarti A Undetected genotyping errors cause apparent overtransmission of common alleles in the transmission/disequilibrium test American Journal of Human Genetics 2003 72 3 598 610 10.1086/368203 12587097 PMC1180236

[B30] Schaid DJ Guenther JC Christensen GB Hebbring S Rosenow C Hilker CA McDonnel SK Cunningham JM Slager SL Blute ML Thibodeau SN Comparison of microsatellites versus single-nucleotide polymorphisms in a genome linkage screen for prostate cancer-susceptibility loci American Journal of Human Genetics 2004 75 6 948 965 10.1086/425870 15514889 PMC1182157

[B31] Ewen KR Bahlo M Treloar SA Levinson DF Mowry B Barlow JW Foote SJ Identification and analysis of error types in high-throughput genotyping American Journal of Human Genetics 2000 67 3 727 736 10.1086/303048 10924406 PMC1287531

[B32] Bjorklund M A method for adjusting allele frequencies in the case of microsatellite allele drop-out Molecular Ecology Notes 2005 5 3 676 679 10.1111/j.1471-8286.2005.00992.x

[B33] Buchan JC Archie EA van Horn RC Moss CJ Alberts SC Locus effects and sources of error in noninvasive genotyping Molecular Ecology Notes 2005 5 3 680 683 10.1111/j.1471-8286.2005.01002.x

[B34] DeWoody J Nason JD Hipkins VD Mitigating scoring errors in microsatellite data from wild populations Molecular Ecology Notes 2006 6 4 951 957 10.1111/j.1471-8286.2006.01449.x

[B35] Morin PA LeDuc RG Archer E Martien KK Taylor BL Huebinger R Bickham JW Estimated genotype error rates from bowhead whale microsatellite data 2007 http://iwcoffice.org/_documents/sci_com/workshops/SC-59-BRG15%28draft%29.pdf Unpublished

[B36] Flagstad O Hedmark E Landa A Brøseth H Persson J Andersen R Segerstrom P Ellegren H Colonization history and noninvasive monitoring of a reestablished wolverine population Conservation Biology 2004 18 3 676 688 10.1111/j.1523-1739.2004.00328.x-i1

[B37] Mowat G Paetkau D Estimating marten Martes americana population size using hair capture and genetic tagging Wildlife Biology 2002 8 3 201 209

[B38] Pearse DE Eckerman M Janzen FJ Avise C A genetic analogue of 'mark-recapture' methods for estimating population size: an approach based on molecular parentage assessments Molecular Ecology 2001 10 11 2711 2718 10.1046/j.0962-1083.2001.01391.x 11883884

[B39] Poole KG Mowat G Fear DA DNA-based population estimate for grizzly bears Ursus arctos in northeastern British Columbia, Canada Wildlife Biology 2001 7 2 105 115

[B40] Prugh LR Ritland CE Arthur SM Krebs CJ Monitoring coyote population dynamics by genotyping faeces Molecular Ecology 2005 14 5 1585 1596 10.1111/j.1365-294X.2005.02533.x 15813796

[B41] Schwartz MK Luikart G Waples RS Genetic monitoring as a promising tool for conservation and management Trends in Ecology & Evolution 2007 22 1 25 33 10.1016/j.tree.2006.08.009 16962204

[B42] Woods JG Paetkau D Lewis D McLellan BN Proctor M Strobeck C Genetic tagging of free-ranging black and brown bears Wildlife Society Bulletin 1999 27 3 616 627

[B43] Christie MR Parentage in natural populations: novel methods to detect parent-offspring pairs in large data sets Molecular Ecology Resources 2010 10 1 115 128 10.1111/j.1755-0998.2009.02687.x 21564996

[B44] McLean JE Seamons TR Dauer MB Bentzen P Quinn TP Variation in reproductive success and effective number of breeders in a hatchery population of steelhead trout (Oncorhynchus mykiss): examination by microsatellite-based parentage analysis Conservation Genetics 2008 9 2 295 304 10.1007/s10592-007-9340-0

[B45] Vandeputte M Mauger S Dupont-Nivet M An evaluation of allowing for mismatches as a way to manage genotyping errors in parentage assignment by exclusion Molecular Ecology Notes 2006 6 1 265 267 10.1111/j.1471-8286.2005.01167.x

[B46] Herbinger C Doyle CT Taggart CT Lochmann SE Brooker AL Wright JM Cook D Family relationships and effective population size in a natural cohort of Atlantic cod (Gadus morhua) larvae Canadian Journal of Fisheries and Aquatic Sciences 1997 54 Suppl 1 11 18

[B47] Prodohl PA Loughry WJ McDonough CM Nelson WS Thompson EA Avise JC Genetic maternity and paternity in a local population of armadillos assessed by microsatellite DNA markers and field data American Naturalist 1998 151 1 7 19 10.1086/286098 18811420

[B48] Okland JM Haaland OA Skaug HJ A method for defining management units based on genetically determined close relatives Ices Journal of Marine Science 2010 67 3 551 558 10.1093/icesjms/fsp260

[B49] Skaug HJ Allele-sharing methods for estimation of population size Biometrics 2001 57 3 750 756 10.1111/j.0006-341X.2001.00750.x 11550924

[B50] Skaug HJ Berube M Palsboll P Detecting dyads of related individuals in large collections of DNA-profiles by controlling the false discovery rate Molecular Ecology Resources 2010 10 693 700 10.1111/j.1755-0998.2010.02833.x 21565074

